# Common mental health problems among patients accessing care at ayurvedic health centers in Udupi Taluk.

**DOI:** 10.12688/f1000research.165056.2

**Published:** 2025-09-15

**Authors:** Surekha D Shetty, Vidya Prabhu, Varalakshmi Chandra Sekaran, Ashwini Aithal p, Sapna Marpalli, Ravichandran Nair K, Shantha Kumari BR

**Affiliations:** 1 Division of Anatomy, Department of Basic Medical Sciences,, Manipal Academy of Higher Education, Manipal, Karnataka, 576104, India; 2 Department of Global Public Health Policy and Governance, Prasanna School of Public Health, Manipal Academy of Higher Education, Manipal, Karnataka, 576104, India; 3 Department of Global Public Health Policy and Governance, Prasanna School of Public Health, Manipal Academy of Higher Education, Manipal, Karnataka, 576104, India; 4 Division of Anatomy, Department of Basic Medical Sciences, Manipal Academy of Higher Education, Manipal, Karnataka, 576104, India; 5 Division of Anatomy, Department of Basic Medical Sciences, Manipal Academy of Higher Education, Manipal, Karnataka, 576104, India; 6 Department of Social and Health Innovation Prasanna School of Public Health, Manipal Academy of Higher Education, Manipal, Karnataka, 576104, India; 7 Division of Pathology, Department of Basic Medical Sciences, Manipal Academy of Higher Education, Manipal, Karnataka, 576104, India

**Keywords:** Ayurveda health centers, holistic well-being, common mental health problems, factors of stress, COVID-19.

## Abstract

**Introduction:**

Increased work pressure, lifestyle modifications, and deteriorated living conditions brought on by Coronavirus disease 2019 (COVID-19) can exacerbate mental health concerns such as anxiety, sleeplessness, depression, and stress-related illnesses. Recent studies have shown that a high burden of mental health conditions, including depression, anxiety disorders, stress, panic attacks, impulsivity, irrational anger, somatization disorder, sleep disorders, emotional disturbance, symptoms of post-traumatic stress disorder, and suicidal thoughts, may be experienced by COVID-19 survivors. Due to differences in their susceptibility to infection and lifestyle factors, people with different medical conditions and social roles may have various kinds and severities of mental health problems. This study aimed to provide essential insights into the prevalence, types, and linkages of various mental health issues to improve patient outcomes, guide future treatments, and advance holistic well-being.

**Methodology:**

This cross-sectional study examined the prevalence of common mental health issues among patients receiving treatment at Ayurvedic health centers in Udupi Taluk. Five Ayurvedic centers were chosen randomly from 275 Ayurvedic centers registered in Udupi Taluk. A total of 423 patients aged 18 years and above were selected for this study. The Perceived Stress Scale and Patient Health Questionnaire (PHQ-9) were used to collect data.

**Results and discussion:**

The study revealed the presence of some level of perceived stress and depression among the participants. The factor associated with stress was the educational status of participants (p =0.01). The comparison between different stress levels revealed that moderate stress levels were significantly higher among participants with a lower educational status.

**Conclusion:**

According to a study on perceived stress levels and educational status, participants with lower educational status reported feeling more stressed than those with higher educational status.

## Introduction

According to the World Health Organization (WHO), health is defined as “a state of complete physical, mental and social well-being and not merely the absence of disease or infirmity”. ^1^ Mental health, therefore, is a vital component of overall health and wellbeing. A healthy mind is an essential aspect of wellness, and during illness, physical limitations can manifestations as psychiatric symptoms, causing patients to experience a wide range of psychological problems. ^2^ This study was conducted to determine the common mental health problems among patients accessing care at Ayurvedic health centers in Udupi taluk.

Common mental health disorders (CMDs), such as depression, anxiety, and stress, are leading contributors to disability worldwide. According to the WHO, one in every eight people worldwide is suffering from mental disorders, with depression and anxiety being among the most prevalent. ^3^ In India, the National Mental Health Survey (2015–16) reported that nearly 14% of the population suffers from mental health problems. ^4^ Despite its high prevalence, access to timely diagnosis and treatment remains limited, especially in nonpsychiatric healthcare settings.

Coronavirus disease 2019 (COVID-19) was characterized as a pandemic by the WHOon March 11, 2020. ^5^ The pandemic significantly increased the burden of CMDs. In 2020, the COVID-19 pandemic led to a sharp increase in mental disorders, with a 26% increase in anxiety disorders and a 28% increase in major depressive disorders within just one year. ^3^ Hundreds of millions of people’s lives have been impacted, and people’s social, occupational, educational, and lifestyle choices have changed due to individual and governmental reactions to this serious public health catastrophe. Worry about infection, increased workload, lifestyle changes, and social isolation contributed to mental health problems such as anxiety, depression, insomnia, and stress-related disorders. ^6– 9^ People with diverse health conditions and social roles are affected differently, depending on their vulnerability to infection and coping mechanisms. ^10^ Studies have also reported a high prevalence of depression, anxiety, posttraumatic stress symptoms, and suicidal ideation among COVID-19 survivors. ^11, 12^ The COVID-19 pandemic, according to the United Nations, not only compromises physical health but also increases psychological distress. ^13^ Therefore, it is crucial to understand how communities’ mental health is affected during this pandemic. According to recent research, COVID-19 survivors may experience a high burden of mental health issues, such as depression, anxiety disorders, stress, panic attacks, irrational anger, impulsivity, somatization disorder, sleep disorders, emotional disturbance, post-traumatic stress symptoms, and suicidal ideation. ^14^ Additional factors associated with mental health problems during COVID-19 include age, gender, marital status, education, occupation, income, place of residence, close contact with COVID-19 patients, comorbid physical and mental health issues, exposure to COVID-19-related news and social media, coping mechanisms, stigma, psychosocial support, health communication, trust in medical services, personal protective measures, and risk of COVID-19 infection. ^15^

In the Indian context, multiple factors, such as age, gender, marital status, education, income, occupation, comorbidities, stigma, and exposure to health-related news, have been associated with CMDs. ^16^ However, much of the existing evidence comes from general or psychiatric healthcare settings, whereas alternative systems of medicine remain underexplored.

Ayurveda, a millennia-old Indian medical system, exemplifies this holistic concept by recognizing the interdependence of mind, body, and spirit. ^17^ Ayurvedic wellness centers have become popular as alternative and complementary healthcare destinations, promising physical and emotional renewal. ^18^

Despite their growing use, there is limited evidence on the prevalence of CMDs among patients seeking care in such centers. Addressing this gap is essential to ensure that mental health is not overlooked in integrative healthcare models.

There is a bidirectional relationship between physical health and mental health. Physical issues can lead to psychological distress, whereas mental health issues can manifest as physical symptoms. ^19^ In the context of Ayurveda, which encourages balance and harmony. Exploring the prevalence of common mental health issues is particularly crucial. Healthcare practitioners, policymakers, and researchers should work together to improve the overall quality of care at Ayurvedic health centers by highlighting this little-studied aspect. Furthermore, the findings of this study may help understand better-integrated healthcare approaches that prioritize mental and physical health holistically.

Given the growing interest in alternative healthcare practices and the global burden of mental health disorders, this study aimed to fill a critical knowledge gap by investigating the common mental health issues encountered by patients seeking care at Ayurvedic health centers.

By highlighting the mental health dimension of care in these holistic settings, the study seeks to inform future strategies to enhance patient outcomes and promote well-being in an integrative, mind-body-centered healthcare framework.

## Methodology

This cross-sectional study was conducted between June 2022 and December 2022 in Udupi Taluk, Karnataka. Five of the 275 Ayurvedic centers registered in the region were selected using simple random sampling.

All patients aged 18 years and above who attended the selected centers during the study period were eligible to participate. Using an anticipated prevalence of 50%, 95% confidence interval, and 5% margin of error, the minimum required sample size was calculated as 384. Considering a 10% nonresponse rate, 423 participants were included in the study through consecutive sampling.

Ethical approval was obtained from the Kasturba Medical College and Kasturba Hospital Institutional Ethics Committee (IEC No: 866--2021) on May 9, 2022. Written informed consent was obtained from all participants prior to data collection.

Data were collected using standardized, validated tools. The Patient Health Questionnaire-9 (PHQ-9) was used to screen for depression symptoms, and the Perceived Stress Scale (PSS) was used to measure stress levels. ^19^ The tool also included COVID-19-related items to capture participants’ perceptions and emotional responses during the pandemic. These items were included to explore contextual factors potentially influencing mental health, given the timing of data collection during the pandemic.

## Results and discussion

Sociodemographic characteristics of the participants are presented in Table 1. Of the 430 participants, 61.6% were in the age group of 31–60 years, and the majority (56%) were male. Most of the participants (56.7%) had less than PUC education. Most participants (78.8%) were married, and 85.5% belonged to the Hindu religion.

**Table 1.** Socio-demographic details of the participants.

Variables	Frequency & percentage
**Age in years**	
18–30	144 (33.5)
31–60	265 (61.6)
More than 61	21 (4.9)
**Gender**	
Male	241 (56)
Female	189 (44)
**Educational status**	
Less than PUC	244 (56.7)
Degree and above	186 (43.3)
**Marital status**	
Single	91 (21.2)
Married	339 (78.8)
**Religion**	
Hindu	368 (85.6)
Muslim	44 (10.2)
Christian	18 (4.2)
**Employment status**	
Employed	297 (69.1)
Unemployed	100 (23.3)
Student	26 (6.0)
Retired	7 (1.6)

Most of the people in the population were between the ages of 31 and 60 (61.6%), followed by people between the ages of 18 and 30 (33.5%), and over 61 (4.9%). There were a slightly higher number of men (56%) than women (44%). Almost half of the people in the country had less than a Pre-University Course (PUC) education (56.7%), while 43.3% had a degree or higher. Seventy-eight percent of people were married, while only twenty-two percent were single. Hindus (85.6%), Muslims (10.2%), and Christians (4.2%). A large part of the population was working (69.1%), while smaller portions were unemployed (23.3%), school (6%), or retired (1.6%). The distribution of demographics revealed the needs and problems of the community.

The prevalence of common mental health problems is presented in Table 2. Regarding stress, most participants (93%) had experienced moderate stress, 5.1% had experienced low stress, and 1.9% had experienced high stress. Regarding depression, 41.4% had experienced minimal depression, 27.9% had experienced moderate depression, 15.8% had experienced mild depression, 14.2% had experienced moderate to severe depression, and 0.7% had experienced severe depression.

**Table 2.** Prevalence of common mental health problems during covid-19 pandemic.

Variable	Frequency (%)
**Stress**	
Low stress	22 (5.1)
Moderate stress	400 (93)
High stress	8 (1.9)
**Depression**	
Minimal depression	178 (41.4)
Mild depression	68 (15.8)
Moderate depression	120 (27.9)
Moderately severe depression	61 (14.2)
Severe depression	3 (0.7)

The associations between common mental health problems and demographic characteristics of the participants are presented in Table 3.

**Table 3.** Association between common mental health problems and demographic characteristics of the participants.

	Low stress	Moderate stress	High stress	p-value
**Age**				
18-30	8 (36.4%)	130 (32.5%)	6 (75.0%)	
31-60	13 (59.1%)	250 (62.5%)	2 (25.0%)	0.1
More than 61	1 (4.5%)	20 (5.0 %)	0 (0.0%)	
**Gender**				
Male	14 (63.6%)	222 (55.5%)	5 (62.5%)	0.7
Female	8 (36.4%)	178 (44.5%)	3 (37.5%)	
**Educational status**				
Less than PUC	7 (31.8%)	236 (59.0%)	1 (12.5%)	
Degree and above	15 (68.2%)	164 (41.0%)	7 (87.5%)	0.001 *
**Employment status**				
Employed	17 (77.3%)	275 (68.8%)	5 (62.5%)	
Unemployed	2 (9.1%)	97 (24.2%)	1 (12.5%)	0.1
Student	2 (9.1%)	22 (5.5%)	2 (25.0%)	
Retired	1 (4.5%)	6 (1.5%)	0 (0.0%)	
**Area of residence**				
Rural	16 (72.7%)	226 (56.5%)	4 (50.0%)	0.3
Urban	6 (27.3%)	174 (43.5%)	4 (50.0%)	

	Minimal depression	Mild depression	Moderate depression	Moderately severe depression	Severe depression	p-value
**Age**						
18-30	58 (32.6%)	23 (33.8%)	42 (35.0%)	21 (34.4%)	0 (0.0%)	
31-60	113 (63.5%)	42 (61.8%)	69 (57.5%)	38 (62.3%)	3 (100.0%)	0.7
More than 61	7 (3.9%)	3 (4.4%)	9 (7.5%)	2 (3.3%)	0 (0.0%)	
**Gender**						
Male	98 (55.1%)	42 (61.8%)	72 (60.0%)	27 (44.3%)	2 (66.7%)	0.2
Female	80 (44.9%)	26 (38.2%)	48 (40.0%)	34 (55.7%)	1 (33.3%)	
**Educational status**						
Less than PUC	115 (64.6%)	35 (51.5%)	61 (50.8%)	31 (50.8%)	2 (66.7%)	
Degree and above	63 (35.4%)	33 (48.5%)	59 (49.2%)	30 (49.2%)	1 (33.3%)	0.3
**Employment status**						
Employed	120 (67.4%)	51 (75.0%)	81 (67.5%)	43 (70.5%)	2 (66.7%)	
Unemployed	44 (24.7%)	14 (20.6%)	24 (20.0%)	17 (27.9%)	1 (33.3%)	
Student	12 (6.7%)	3 (4.4%)	10 (8.3%)	1 (1.6%)	0 (0.0%)	0.3
Retired	2 (1.1%)	0 (0.0%)	5 (4.2%)	0 (0.0%)	0 (0.0%)	
**Area of residence**						
Rural	107 (60.1%)	43 (63.2%)	63 (52.5%)	31 (50.8%)	2 (66.7%)	0.4
Urban	71 (39.9%)	25 (36.8%)	57 (47.5%)	30 (49.2%)	1 (33.3%)	

* Statistical significance at p < 0.05.

### Stress distribution across age groups

The data suggest that the highest percentage (75%) of high stress is found among the youngest age group (18-30 years), and factors such as career uncertainty, financial instability, or life transitions may contribute to higher stress levels in younger individuals, whereas the middle-aged group (31-60 years) has a lower percentage (25%) of high stress, which may be due to a decrease in stress with age aligned with possible increases in life experience, stability, or coping mechanisms.

No participants over 61 years reported high stress, which may be influenced by retirement and reduced occupational pressure. Statistical analysis revealed no significant association between age and stress level (p = 0.1).

### Impact of educational status on stress levels

A statistically significant association was found between education status and stress (p = 0.001). Among participants with less than a PUC education, 59% experienced moderate stress, whereas 87.5% of those with a degree or higher reported high stress. People with a bachelor’s degree or higher may be more stressed because they have more responsibilities, higher career demands, or work pressures. These findings suggest that educational attainment influences stress perception and experience, which could inform targeted mental health interventions.

### Stress distribution across employment status

The data show that people with different types of jobs experience various levels of stress. The employed individuals experienced moderate stress (68.8%), and approximately 24.2% of the unemployed people were in the moderate stress category. Notably, the students were in the high-stress category (25%). People with jobs usually feel moderate rather than high or low stress because of pressures at work, such as meeting deadlines, worrying about job stability, balancing work and life, and meeting performance standards.

Stress distribution across rural and urban areas:

The data revealed that 72.7% of the participants from rural areas reported low stress, whereas 27.3% were from urban areas. For moderate stress, 56.5% of the participants were from rural areas, and 43.5% were from urban areas. In the high-stress category, the distribution was equal, with 50% of the participants from rural areas and 50% from urban areas.

Although these figures suggest that rural participants were more likely to report lower levels of stress, differences in stress levels between rural and urban populations were not statistically significant.

The participants’ feelings and thoughts during the COVID-19 pandemic are presented in Table 4. Approximately one-third of the participants (34.2%) were extremely afraid, while 33.7% were rarely afraid of acquiring COVID-19 when going into the public. Nearly half (49.8%) of the participants were worried about contracting COVID-19. Approximately 41.6% of the participants reported that sleep was rarely affected because of thoughts related to COVID-19. About 22.3% reported avoiding conversations on COVID-19 because of fear or anxiety. Approximately 36.7% were worried about acquiring COVID-19 when an unknown person came closer to them. Approximately 20.9% were anxious about obtaining information on COVID-19. Approximately 40.5% were concerned about acquiring COVID-19 when people coughed or sneezed.

**Table 4.** Participants’ feelings and thoughts during COVID-19.

Characteristics	Frequency (%)
**Afraid of acquiring COVID-19 when going into the public**	
Extremely Afraid	147 (34.2)
Sometimes Afraid	113 (26.3)
Rarely Afraid	145 (33.7)
Not at All Afraid	25 (5.8)
**Feeling worried of acquiring COVID-19**	
Always	65 (15.1)
Sometimes	214 (49.8)
Rarely	120 (27.9)
Never	31 (7.2)
**Sleep get affected because of thoughts relating COVID-19**	
Always	85 (19.8)
Sometimes	122 (28.4)
Rarely	179 (41.6)
Never	44 (10.2)
**Avoiding conversations on COVID-19 out of fear/anxiety**	
Always	96 (22.3)
Sometimes	130 (30.2)
Rarely	165 (38.4)
Never	39 (9.1)
**Worried acquiring COVID-19 when unknown person coming close**	
Extremely Afraid	158 (36.7)
Sometimes Afraid	106 (24.7)
Rarely Afraid	147 (34.2)
Not at All Afraid	19 (4.4)
**Anxious when knowing information on COVID-19**	
Extremely Afraid	90 (20.9)
Sometimes Afraid	138 (32.1)
Rarely Afraid	167 (38.8)
Not at All Afraid	35 (8.1)
**Concerned of acquiring COVID-19 when people cough or sneeze**	
Extremely Afraid	174 (40.5)
Sometimes Afraid	70 (16.3)
Rarely Afraid	170 (39.5)
Not at All Afraid	16 (3.7)

## Discussion

The present study revealed the prevalence of common mental health disorders among patients seeking care at Ayurvedic health centers during the COVID-19 pandemic.

Rather than directly attributing these findings to COVID-19, it is important to recognize that the cross-sectional design captures participants’ mental health only at a single point in time and does not establish causality. While the pandemic may have been one of several contextual stressors influencing participants, other factors, such as preexisting health conditions, ongoing treatments, and the stress of current illness, could also have contributed to their perceived stress and depressive symptoms.

### Pandemic-induced stressors

People who took part were probably more stressed because they were always worried about their health, the health of their loved ones, and the chance of contracting the virus. A significant source of stress was worry that they would get sick and have to go to the hospital or die. Many people lost their jobs, had their incomes dropped, or were unsure of where to work in the future because of the pandemic. This lack of financial security probably stressed people, especially those who were already weak. Measures to keep people from getting too close, lockdowns, and limits on meetings all messed everyday social interactions, making people feel lonely, isolated, and disconnected. Not having friends or family during these times could have worsened the worry. People may have been too stressed by the confusing or scary information that kept coming in about the virus, how it spread, and how to stop it. The constant need to handle new information and adjust to new rules may have stressed people.

However, these influences should be interpreted as potential background factors rather than definitive causes of the observed stress levels in this study.

### Psychological impact of prolonged uncertainty

Long-lasting uncertainty about how long the pandemic would last, how well vaccines would work, and the chance of new strains added to stress. Living in a state of long-term uncertainty can be mentally draining and make you feel powerless or burnt out. Significant mental and emotional changes had to be made to deal with sudden and ongoing changes in daily routines, such as working from home or teaching, or changes in social behavior, such as wearing masks and avoiding public places. Getting used to these new rules likely made the volunteers feel more stressed. The fact that many of the people who took part in the study reported moderate amounts of stress suggests that, while not everyone was stressed out, a large part of the population was. There are many signs of mild stress, such as irritability, trouble concentrating, changes in sleep habits, or a general feeling of unease. Moderate stress was expected, which could mean that people were using ways to deal with it, like sticking to routines, getting social support, or doing things that relieve stress. However, these methods are not always sufficient to entirely reduce the stress caused by the pandemic. Different levels of stress among participants could show various levels of vulnerability and resilience. People’s mental health problems, ability to receive social support, ability to handle stress, and coping skills may all affect how they feel and deal with stress during the pandemic.

### Mental health interventions

Many people feel moderate worry, which shows how important it is to have easy access to mental healthcare during and after the pandemic. Helping people deal with long-term stress sources could come from counselling, workshops on stress management, and neighborhood support groups. When people talk about public health, they should focus on physical and mental health, giving them tools and tips for dealing with stress. This could mean encouraging people to live healthy lives, make friends, and obtain clear, consistent knowledge to lower uncertainty. Having moderate worry over a long period could lead to more serious mental health problems such as depression or anxiety if not dealt with. It is essential to keep an eye on and help people with mental health problems as part of the overall reaction to the pandemic. Realizing that many people are under moderate stress clarifies how important it is to build group resilience. Communities are vital for helping each other, creating a sense of unity, and sharing tools to deal with stress in a group. When making decisions, policymakers should consider how the pandemic affects people’s mental health, and ensure that mental health services are adequately paid and included in plans for dealing with the pandemic. This includes helping people who may be more influenced than others, such as frontline workers, people with low incomes, and people who already have mental health problems.

The study result is aligned with the study conducted in Kerala, which also reported that most of the participants had experienced moderate levels of stress during COVID–19. ^21^ In the current study, stress was high among participants aged 31 to 60. Stress levels were low among participants over 60 years of age. Although the finding was not statistically significant, previous research and global perceived stress reported that stressors experienced by older people are lower than those of younger people. ^22^ The association between gender and perceived stress showed that the moderate level of stress was higher among males than compared to females. This finding contradicts previous literature that reported lower perceived stress levels in men. ^23, 24^

A comparison between educational status and perceived stress level revealed that perceived stress was higher among participants with lower educational status than among those with higher educational status. The comparison between different levels of stress revealed that moderate levels of stress were higher among participants with lower educational status, and this was found to be statistically significant.

Education and stress resilience:

People with more schooling usually have better access to cognitive resources and ways to deal with problems. People who attend college often learn how to solve problems, think critically, and control their emotions, which makes them better able to handle stress. Higher education levels usually improve people’s ability to find, understand, and use health knowledge, which can help them better deal with stress. They may also have more knowledge about ways to deal with stress, mental health tools, and self-care activities that can help them feel less stressed. A college degree is often linked to better job opportunities, higher income, and more stable finances, which can help lower financial stress, which is a major cause of overall stress. Financially stable people can focus on their health and happiness instead of the day-to-day worries that people with less schooling might have. People with lower levels of education may not have many ways to deal with these problems. They may not know how to handle stressful situations, making them feel more stressed, especially during tough times such as a pandemic. A lower level of education is often linked to jobs that pay less, job insecurity, and less access to healthcare and other helpful tools. Being economically vulnerable is a significant source of stress because it can make people worry about meeting their basic needs, especially during crises, such as the COVID-19 pandemic. People with less schooling may also have to deal with extra social and environmental stresses, such as living in an unsafe or crowded place, not having much social support, and being discriminated against or socially stigmatized. These factors can worsen the levels of worry. The result that people with less education had higher moderate stress levels was statistically significant. This shows a strong link between the level of education and the ability to deal with or understand stress, with less education linked to higher stress levels. Even though moderate stress is not as bad for you as high stress, it can still significantly affect your health and wellbeing. People with less schooling may be more likely to experience moderate stress because they have more trouble with money, fewer friends and family, and less access to mental health resources.

### Implications for interventions

Based on these results, it is clear that people with less education require specific support services. Some examples of interventions include classes on how to deal with stress, handle money, and obtain affordable mental health services. Promoting higher education and learning opportunities throughout life can help people deal with stress. Even as an adult, encouraging and helping people keep learning could help vulnerable groups feel less stressed. When planning mental health interventions, public health plans should consider the educational differences. Messages and support services tailored to the needs of people with less schooling can help to close the gap in their ability to deal with stress.

Policy implications:

Reducing educational inequality can help people feel less stressed and better about their general health in the long term. Stress differences could be lessened by policies that ensure equal access to good schools, job training, and adult education. A significant social factor affecting health is education level. Policymakers should consider how education affects health, such as stress, and work to lower hurdles to education, especially in areas that do not receive enough help. Obtaining help and tools for people with less education through community-based programs can be very helpful in lowering stress. Community centers may offer educational classes, counselling for mental health issues, and social support networks as part of these efforts.

A comparison between employment status and perceived stress level revealed that perceived stress was higher among employed participants, although the association was not statistically significant. A comparison between different levels of perceived stress reported that moderate levels of perceived stress were higher among employed participants. As compared to area of residence the rural participants experienced more perceived stress as compared to urban participants. Except for educational status, none of the factors were found to be statistically significant.

The present study revealed that some levels of depression were exhibited by participants during COVID-19. Depression was high among the participants aged 31–60 years. Stress levels were low among participants over 60 years of age. The association between gender and PHQ-9 depression score was higher among males than among females. A comparison between educational status and PHQ-9 scores showed that depression was higher among participants with a lower educational status than among those with a higher educational status. The comparison between employment status and PHQ-9 revealed that depression was higher among employed participants. Depression was higher among rural participants than among urban participants. None of the factors were statistically significant.

## Conclusion

This study aimed to determine the prevalence of common mental health problems among patients accessing care at Ayurvedic health centers during the COVID-19 pandemic. The study revealed the presence of varying level of perceived stress and depression among the participants. The reasons for perceived stress and depression, and effective measures to overcome these problems need to be explored. The further research needs to be carried out to investigate the reasons and measures to overcome these problems. This may help to overcome stress and depression during crises.

### Limitations

The cross-sectional design prevents the establishment of causal relationships between COVID-19 and observed stress and depression levels. The study did not differentiate between patients with or without a COVID-19 diagnosis and did not control for potential confounders such as pre-existing health conditions. These factors may have independently influenced participants’ mental health outcomes. Therefore, the results should be interpreted as an assessment of mental health status in the context of the pandemic rather than as direct consequences of COVID-19 itself.

## Ethical approval and consent

The approval has been obtained from Kasturba Medical College and Kasturba Hospital Institutional Ethics Committee (IEC No: 866-2021), and written consent from the participants was also obtained. The ethical clearance was obtained on May 9, 2022.

## Data Availability

Figshare: Common mental health problems among patients accessing care at ayurvedic health centers in Udupi Taluk https://doi.org/10.6084/m9.figshare.29320637.v2 ^25^ The project contains the following underlying data:
• Mental stress.xls. (Anonymised answers to questionnaire). Data are available under the terms of the Creative Commons Attribution 4.0 International license (CC-BY 4.0). Figshare: Common mental health problems among patients accessing care at ayurvedic health centers in Udupi Taluk https://doi.org/10.6084/m9.figshare.29320637.v2 ^25^ The project contains the following data:
• Questionnaire.pdf Data are available under the terms of the Creative Commons Attribution 4.0 International license (CC-BY 4.0).
