# Supplementary material for: Common mental health problems among patients accessing care at ayurvedic health centers in Udupi Taluk
Source: F1000Res. 2025 Sep 15;14:749. Originally published 2025 Jul 30. [Version 2] doi: 10.12688/f1000research.165056.2 (PMC12635521; doi:10.12688/f1000research.165056.2)
Supplement: Peer review history

# Reviewer report: Reviewer response for version 2

**DOI:** 10.5256/f1000research.187956.r428982

**Published:** 2025-11-20

**Authors:** Ankur Kumar Tanwar

**Affiliations:** 1 Kriya Sharir, Divya Jyoti Ayurvedic Medical College and Hospital, Sarita Vihar, New Delhi, India

1. Author should add COVID-19 in title as the study focused on mental health issue after COVID-19.

 2. Considering that the title highlights a common mental health issue, it is essential for the author to also explore additional mental health concerns that may be interconnected.

 3. The methodology section should specify the statistical test employed and provide a clear description of the exclusion criteria.

 4. The discussion section should incorporate additional references

Is the work clearly and accurately presented and does it cite the current literature?

Partly

If applicable, is the statistical analysis and its interpretation appropriate?

Yes

Are all the source data underlying the results available to ensure full reproducibility?

Yes

Is the study design appropriate and is the work technically sound?

Partly

Are the conclusions drawn adequately supported by the results?

Yes

Are sufficient details of methods and analysis provided to allow replication by others?

Partly

Reviewer Expertise:

Physiology

I confirm that I have read this submission and believe that I have an appropriate level of expertise to confirm that it is of an acceptable scientific standard, however I have significant reservations, as outlined above.

# Reviewer report: Reviewer response for version 2

**DOI:** 10.5256/f1000research.187956.r428988

**Published:** 2025-11-13

**Authors:** Sangeeth Somanadhapai

**Affiliations:** 1 Shree Guru Gobind Singh Tricentenary University, Gurugram, Haryana, India

Summary

 Cross-sectional survey of patients attending five randomly selected Ayurvedic centres in Udupi Taluk (June–Dec 2022) using the PHQ-9 and Perceived Stress Scale (PSS) to estimate prevalence and investigate associations between stress/depression and sociodemographic factors. Sample size reported as 423 (calculated from 384 + 10% nonresponse). Major Strengths

- Topical and understudied setting: Studying mental health in patients attending Ayurvedic centres addresses a real gap in integrative/alternative care contexts.
- Use of validated instruments & open data: The PHQ-9 and PSS are established tools; the anonymised dataset and questionnaire are shared on Figshare (good reproducibility practice).
- Ethics documented: Institutional Ethics Committee approval and written informed consent are clearly stated (IEC No: 866-2021).

Major Concerns

- Inconsistency in reported sample size / participant count: Methods mention 423 participants but Results state 430; needs clarification and explanation of exclusions or errors.
- Extremely skewed stress distribution: 93% moderate stress is highly unusual — authors should specify version, scoring, and categorization of PSS and include descriptive stats for raw scores.
- Statistical methods insufficiently described: Tests used are unclear; report exact methods, effect sizes, confidence intervals, and consider multivariable analysis.
- Sampling & representativeness: Discuss potential cluster sampling bias; justify 5-centre selection, show prevalence by centre, and clarify representativeness.
- Confounding and interpretation: Avoid causal language, discuss unmeasured confounders, and address possible selection bias.
- Multiple testing & selective reporting: Address risk of type I error; pre-specify hypotheses or adjust for multiple comparisons.

Minor Concerns / Presentation Issues

- Ensure consistency in tables (totals, percentages) and fix all formatting issues.
- Provide statistical software details, test names, and missing-data handling methods.
- Add comparison with similar Indian or international studies for context.
- Add figures (histogram or boxplots for PSS and PHQ-9 scores).
- Improve typographical consistency (spacing, percentage notation, etc.).

Concrete, Actionable Suggestions

- Resolve N discrepancy and provide participant flow diagram.
- Report raw instrument data with mean, SD, median, IQR; specify cutoffs and references.
- Clearly state statistical tests, software, alpha level, and multiple comparison handling.
- Discuss or account for clustering (centre-level analysis).
- Revise discussion for causal tone and add limitation details.
- Improve tables with N, test statistics, and clear p-value alignment.
- Add data visualization plots for key scales.

Recommendation

 Major revision required. The study is relevant and can be potentially indexed after clarifying sample details, improving statistical rigor, addressing design limitations, and enhancing transparency in data presentation.

Is the work clearly and accurately presented and does it cite the current literature?

Yes

If applicable, is the statistical analysis and its interpretation appropriate?

I cannot comment. A qualified statistician is required.

Are all the source data underlying the results available to ensure full reproducibility?

Partly

Is the study design appropriate and is the work technically sound?

Partly

Are the conclusions drawn adequately supported by the results?

Yes

Are sufficient details of methods and analysis provided to allow replication by others?

Yes

Reviewer Expertise:

NA

I confirm that I have read this submission and believe that I have an appropriate level of expertise to state that I do not consider it to be of an acceptable scientific standard, for reasons outlined above.

# Reviewer report: Reviewer response for version 1

**DOI:** 10.5256/f1000research.181650.r403575

**Published:** 2025-08-23

**Authors:** Prafulla Shriyan

**Affiliations:** 1 Public Health Foundation of India, New Delhi, India

The article summarizes the common mental health disorders among patients accessing care at selected Ayurvedic hospitals during Covid-19 pandemic. I appreciate the effort of authors in their interest on assessing mental health disorders among patients seeking care at the health facility. Although the topic was interesting I found major concerns associated with the article. Here is a consolidated comments attached below.

**Introduction and Rationale: **The introduction lacks a logical flow, with disconnected paragraphs that do not build a cohesive argument. It fails to provide sufficient background evidence on the prevalence of common mental health disorders (CMDs) and their contributing factors in the study population, which is essential for establishing context. Furthermore, the study location is inappropriately placed in the introduction rather than the methodology section.

**Methodological Weaknesses: **The methodology section is notably brief and lacks critical details. It does not adequately describe the activities undertaken during the study, nor does it specify the standard tools or instruments used to assess mental health disorders, exposures, and outcomes. A crucial omission is the absence of a clear explanation of the sample size and the justification for its selection.

**Data Analysis and Presentation of Results: **The results section is overly descriptive and could be simplified by focusing on the most significant findings rather than detailing every variable. The authors have not appropriately categorized the stress/depression b Additionally, data presented in Table 4 appears to be entirely new information, as it was not mentioned or justified within the methodology section.

 Inferences and Confounding Factors: The most critical concern is the absence of logic when attributing the findings to COVID-19. The study's cross-sectional design only captures the patients' mental health at a single point in time. The method section does not distinguish them are Covid-19 patients or other patients. It fails to account for other potential confounding factors, such as the patients' pre-existing health conditions and its relationship with the stress of their current illness, all of which could independently contribute to their mental state. Therefore, the statement that the observed stress and depression levels were a direct result of COVID-19 is an unfounded inference that is not supported by the study's design.

Is the work clearly and accurately presented and does it cite the current literature?

Yes

If applicable, is the statistical analysis and its interpretation appropriate?

Partly

Are all the source data underlying the results available to ensure full reproducibility?

Yes

Is the study design appropriate and is the work technically sound?

Yes

Are the conclusions drawn adequately supported by the results?

Partly

Are sufficient details of methods and analysis provided to allow replication by others?

No

Reviewer Expertise:

Mental health disorders, Environmental health, maternal and child health and Nutrition

I confirm that I have read this submission and believe that I have an appropriate level of expertise to confirm that it is of an acceptable scientific standard, however I have significant reservations, as outlined above.

# Response

**Published:** 2025-09-09

**Authors:** Vidya Prabhu

**Affiliations:** Prasanna School Of Public Health, Manipal Academy Of Higher Education, Manipal, India, India

**Response to the reviewer:**

 The article summarizes the common mental health disorders among patients accessing care at selected Ayurvedic hospitals during Covid-19 pandemic. I appreciate the effort of authors in their interest on assessing mental health disorders among patients seeking care at the health facility. Although the topic was interesting I found major concerns associated with the article. Here is a consolidated comments attached below.

**1. Comment**

**Introduction and Rationale: **The introduction lacks a logical flow, with disconnected paragraphs that do not build a cohesive argument. It fails to provide sufficient background evidence on the prevalence of common mental health disorders (CMDs) and their contributing factors in the study population, which is essential for establishing context. Furthermore, the study location is inappropriately placed in the introduction rather than the methodology section.

**Response:**

*We thank for your valuable feedback. We have revised the introduction to improve the logical flow and cohesiveness by restructuring the paragraphs. We have also included information on the prevalence of CMDs. Regarding the study location, we have moved this information from the introduction to the methodology section as suggested, ensuring that the introduction now focuses only on the rationale and background for the study.*

**2.Comment**

**Methodological Weaknesses: **The methodology section is notably brief and lacks critical details. It does not adequately describe the activities undertaken during the study, nor does it specify the standard tools or instruments used to assess mental health disorders, exposures, and outcomes. A crucial omission is the absence of a clear explanation of the sample size and the justification for its selection.

**Response:**

*We thank the reviewer for this valuable comment. In the revised version, we have expanded this section to clearly describe the activities undertaken during the study, including the participant recruitment, data collection and analysis. We have also specified the standard tools used to assess mental health disorders, Furthermore, we have now provided a clear explanation of the sample size calculation, including the assumed prevalence, confidence level, and margin of error.*

**3.Comment**

**Data Analysis and Presentation of Results: **The results section is overly descriptive and could be simplified by focusing on the most significant findings rather than detailing every variable. The authors have not appropriately categorized the stress/depression Additionally, data presented in Table 4 appears to be entirely new information, as it was not mentioned or justified within the methodology section.

**Response:**

*We thank the reviewer for this valuable feedback. We have revised the Results section to highlight the most significant findings and avoid excessive descriptive detail, thereby improving clarity and readability. Regarding the categorization of stress/depression, we have now restructured the presentation and aligned it with the methodology to ensure consistency. Also, we recognize the concern about Table 4. W*e have revised the Methods section and added explanation its inclusion.

**4. Comment**

**Inferences and Confounding Factors:** The most critical concern is the absence of logic when attributing the findings to COVID-19. The study's cross-sectional design only captures the patients' mental health at a single point in time. The method section does not distinguish them are Covid-19 patients or other patients. It fails to account for other potential confounding factors, such as the patients' pre-existing health conditions and its relationship with the stress of their current illness, all of which could independently contribute to their mental state. Therefore, the statement that the observed stress and depression levels were a direct result of COVID-19 is an unfounded inference that is not supported by the study's design.

**Response:**

*We sincerely thank the reviewer for highlighting this important limitation. We agree that the cross-sectional design restricts causal inference, and therefore, it is not possible to attribute stress and depression solely to COVID-19 and acknowledged in the Limitations section that factors such as pre-existing health conditions and concurrent illness may have influenced participants’ mental health status. We believe these revisions improve the scientific accuracy and transparency of the manuscript.*

# Reviewer report: Reviewer response for version 1

**DOI:** 10.5256/f1000research.181650.r407185

**Published:** 2025-08-22

**Authors:** Pankaj Sharma

**Affiliations:** 1 Department of Rejuvenative and Reproductive Medicine in Ayurveda, KLE University's Shri B M Kankanawadi Ayurveda Mahavidyalaya, Belagavi, Karnataka, India

It is a clearly and accurately written and presented paper. Need to consider a few points and incorporate them into the methodology part:

 1. What complaints patients presented to Ayurvedic health centres are not mentioned, and how only mental problems were taken into account or sorted, needs to be clarified.

 2. Whether any Ayurveda interventions were given and if they caused any changes could be a point of discussion in the methodology.

 A few more points:

 1. The paper mentions " **some level of perceived stress**" — the idea is not clear.

 2. Keywords — it would be better if in alphabetical order.

 Apart from that, it is an informative and appropriate study.

Is the work clearly and accurately presented and does it cite the current literature?

Yes

If applicable, is the statistical analysis and its interpretation appropriate?

Yes

Are all the source data underlying the results available to ensure full reproducibility?

Yes

Is the study design appropriate and is the work technically sound?

Yes

Are the conclusions drawn adequately supported by the results?

Yes

Are sufficient details of methods and analysis provided to allow replication by others?

Partly

Reviewer Expertise:

Ayurveda, Indian Medicine, Rasayana and Vajikarna, Rejuvenative and Reproductive Medicine in Ayurveda, Marma Chikitsa, Clinical Nutrition, Ayurveda Psychology and Psychotherapy

I confirm that I have read this submission and believe that I have an appropriate level of expertise to confirm that it is of an acceptable scientific standard.

# Response

**Published:** 2025-09-09

**Authors:** Vidya Prabhu

**Affiliations:** Prasanna School Of Public Health, Manipal Academy Of Higher Education, Manipal, India, India

The suggested changes are incorporated
